# Does the digital economy improve comprehensive total factor productivity in China?

**DOI:** 10.3389/fpubh.2024.1352754

**Published:** 2024-06-14

**Authors:** Guifang Li, Dongdong Ma

**Affiliations:** ^1^College of Economics and Management, Henan Agricultural University, Zhengzhou, China; ^2^School of Economics, Henan University of Economics and Law, Zhengzhou, China

**Keywords:** digital economy, technological innovation, factor endowment structure, human capital, comprehensive total factor productivity, mediating effect model

## Abstract

Total factor productivity is an important symbol of high-quality economic development. At present, the question of whether the digital economy can infuse fresh impetus into enhancing total factor productivity has emerged as a prominent concern in China. This paper constructs a new undesirable output to measure comprehensive total factor productivity (CTFP) with the slack-based measure (SBM) undesirable Malmquist-Luenberger index by using 2011-2020 Chinese provincial panel data. Then, this paper explores the impact of the digital economy (DIG) on CTFP with a fixed effects (FE) panel model and a mediating effect model. The results show that CTFP increases by an average of 3.9%, technical efficiency contributes -1.1%, and the contribution rate of technological progress is 5.0%. Technological progress is the main source of CTFP growth. The empirical findings show that the DIG has a positive and significant impact on CTFP. This paper conducts various robustness tests, and the results remain consistent with the previous conclusion. Moreover, mechanism tests suggest that the promoting effect of the DIG on CTFP can be attributed to three main effects: technological innovation, the factor endowment structure and the educational level. Furthermore, the results of heterogeneity analysis demonstrate that the promoting effect of the DIG on CTFP exists in China’s eastern, central and western regions. The findings of this research can serve as a valuable reference for informing decision-making processes related to environmental governance and high-quality economic development in China.

## 1 Introduction

Since the 1970s, China’s annual economic growth of 10% has been accompanied by environmental pollution, ecological degradation and an excessive consumption of resources (1, 2). However, the report of the 20th National Congress of the Communist Party of China pointed out that promoting green and low-carbon economic and social development is a key link in achieving high-quality development. As an important engine of economic growth, total factor productivity incorporating environmental factors has increasingly become a core indicator in measuring the quality of national or regional economic growth (3, 4). This paper refers to total factor productivity in terms of comprehensive total factor productivity (CTFP), which is the measure of total factor productivity when the comprehensive environmental pollution index is included as an undesirable output.

At present, many countries worldwide regard improving the level of digital economy (DIG) development as one of the paths for high-quality economic development, and China is one such country. The “White Paper on the Development of China’s Digital Economy (2023)” pointed out that the scale of the DIG reached 50.2 trillion RMB in 2022, accounting for 41.5% of GDP, and the DIG has made an enormous contribution to the growth of China’s national economy. The DIG has distinctive features, such as high speed, high innovation, low cost, low pollution, replicability, and shareability. With data elements as the core, the DIG has played a critical role in stimulating consumption and investment and in promoting technological change. The production link has greatly changed the mode of production and consumption as well as people’s way of life, and the DIG is a new engine for improving the efficiency of overall social resource allocation (5, 6).

The literature on total factor productivity measurement with data envelopment analysis (DEA) is mainly divided into two types. On the one hand, some authors treat environmental pollution factors as input factors, but this method does not conform to the actual production process (7, 8). On the other hand, other scholars treat environmental pollution as an undesirable output to measure total factor productivity. The main difference is the choice of the elasticity of environmental pollution variables, such as an environmental pollutant (9, 10), two environmental pollutants (11, 12), three types of industrial waste (13), and a variety of environmental pollutants (14). The research above has not reached a unified conclusion; if the undesirable outputs (the selected environmental pollutants) are different, the measurement results of total factor productivity will also be different.

The academic community presents three different views on the impact of DIG development. First, some scholars believe that digital transformation promotes the economic benefits of real enterprises by reducing costs, improving asset utilization efficiency, and enhancing innovation capabilities. The DIG is characterized by low energy consumption and low pollution emissions. Meanwhile, the characteristics of reproducibility and shareability make data resources overcome the limitations of the traditional law of diminishing marginal returns. The DIG may also reduce environmental pollutants in various ways, thereby improving total factor productivity and ultimately promoting high-quality economic development (15–18). Second, the DIG may have a negative impact on total factor productivity. The application of artificial intelligence technology has a complex effect on total factor productivity. An overreliance on artificial intelligence technology will inhibit total factor productivity growth. Meanwhile, digitization has intensified the competition among enterprises and increased the cost pressure. Some studies have pointed out that the excessive use of artificial intelligence technology will have a certain substitution effect on low-end labor, resulting in an improper allocation of capital and labor and thus damaging production efficiency (19, 20). A third view holds that there is no linear effect between the DIG and total factor productivity. In the early stage of economic development, the technological progress brought by the DIG causes enterprises to reset their production equipment and increase their production by increasing resource extraction and energy consumption. Information technology and the real economy are not fully integrated, and the effect of the DIG cannot be effectively released (21, 22). However, most current studies focus on the impacts of the DIG on total factor productivity, with the combined impact being neglected.

The marginal contributions of this research are mainly as follows. First, this paper constructs a comprehensive environmental pollution index as a new undesirable output to measure CTFP in China with the slack-based measure (SBM) undesirable Malmquist–Luenberger index. Second, this paper clarifies the mechanisms of the direct and indirect effects of the DIG on CTFP, which is a useful supplement to existing research. Third, to explore the specific ways in which the DIG affects CTFP, a mediating effect model including technological innovation, the factor endowment structure and human capital is constructed to explore the transmission mechanisms.

**Figure 1:**
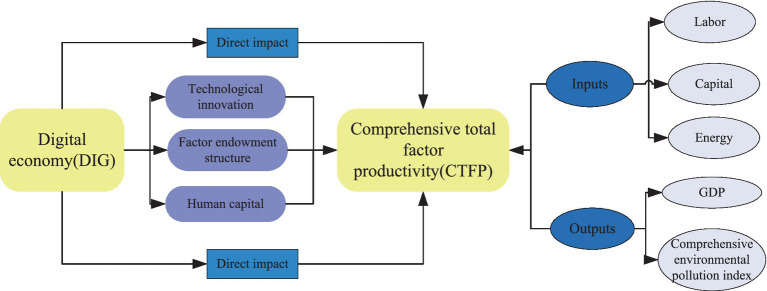
The technical roadmap.

The remainder of this study is organized as follows. Section 2 proposes the theoretical analysis and hypotheses. Section 3 presents the research design, including the samples, data, variables, and methods. Section 4 discusses the empirical results. Section 5 draws the conclusions and policy implications. The technical roadmap of this study is shown in Figure 1.

## 2 Theoretical analysis and hypothesis development

### 2.1 Direct impact of the dig on CTFP

The theoretical logic based on which the DIG drives up CTFP is mainly reflected in the three important aspects. First, the DIG is a new economic model that expands and extends the boundaries of production possibility, changes the constraints of the scale economy under the traditional model, makes new assumptions regarding factors such as labor and capital, and reduces the excessive consumption of factors such as resources and the environment, which ultimately improves the production efficiency of enterprises (4). Second, the DIG itself has spawned a series of innovative technologies, such as artificial intelligence and cloud computing, which have minimized the intermediate links of transactions and reduced transaction costs. At the same time, digital platforms and data analysis can enhance the interaction between producers and consumers, alleviate the mismatch between supply and demand, and thus improve CTFP (23). Third, the traditional economic development model characterized by energy depletion and environmental degradation will lead to high pollution and high energy consumption, which is contrary to the connotation of high-quality economic development. Data elements in the DIG can replace traditional labor and capital, reduce environmental pollution and energy consumption, and are one of the important ways to improve total productivity (16). Therefore, based on the analysis above, this paper proposes the following hypothesis:

*H*1: The DIG will improve CTFP.

### 2.2 Mechanisms of the effect of the dig on CTFP

The digital economy, as a permeable factor, not only directly impacts overall green productivity but also potentially exerts an indirect influence. Solow (24) defined total factor productivity as the economic growth rate after accounting for labor and capital contributions. Technological innovation, factor endowment structure, and human capital are considered crucial factors affecting total factor productivity. Additionally, DIG may impact CTFP through other unobservable channels. Due to data availability and the definition of total factor productivity, this paper focuses on three specific channels through which DIG affects CTFP: technological innovation, factor allocation structure, and human capital.

#### 2.2.1 Technological innovation effect

Technological innovation holds great significance for improving CTFP. Technological innovation plays an important role in environmental protection and sustainable development and provides a valuable way to reduce carbon emissions (25). Big data technology can track the flow of financial resources, weaken adverse selection and moral hazard, promote the output of innovative achievements, realize process re-innovation, transform polluting enterprises with high losses and low output, and realize pollution reduction and carbon reduction. Furthermore, big data, cloud computing, etc., can conduct for an integrated analysis of green product information and consumption preferences, which not only directly improves the production capacity of enterprises but also reduces the energy consumption per unit product by improving the energy efficiency of products and processes. Digital technology can also help make manufacturers’ innovation be data driven, improve the efficiency of green technology research and development (R&D) decision-making, and effectively reduce environmental pollution and carbon emissions (26). Hence, this paper proposes the following hypothesis:

*H*2: The DIG promotes CTFP by driving technological innovation.

#### 2.2.2 Factor endowment structure effect

The DIG may promote CTFP by optimizing the factor endowment structure. The emergence of DIG platforms can effectively alleviate the information asymmetry problem in the market, effectively reducing search, matching, transaction and replication costs (27). Furthermore, the DIG accelerates the cross-regional flow of production factors, leading to the gradual transfer of production factors to enterprises or departments with high productivity and high marginal returns. In particular, it guides the flow and concentration of production factors to green and low-carbon enterprises under the background of increasingly stringent environmental regulation, improves the efficiency of resource allocation, promotes the transformation of resource-consuming enterprises to technological innovation-driven enterprises, and ultimately improves total factor productivity (28). Hence, this paper proposes the following hypothesis:

*H*3: The DIG promotes CTFP by driving the factor endowment structure.

#### 2.2.3 Human capital effect

The development of the DIG has enabled online platform activities to continuously supplement offline agglomeration activities, which not only overcomes the geographical limitations of offline human capital agglomeration but also maintains the online agglomeration activities of human capital. The industrial agglomeration and coordinated development driven by the DIG attract more high-tech enterprises and high-skilled talent, and the resulting scale economies can further reduce the R&D costs of enterprises and promote an improvement in the level of innovation and R&D. Digital platforms have accelerated the spillover and diffusion of cutting-edge technologies in the industrial network, ultimately improving total factor productivity (4). Digital platforms bring a rapid flow of human capital, which can effectively guide the operation of material capital so that high-tech material capital can match the high-level labor force. Human capital can greatly affect technology R&D and application to promote the development of productivity and an improvement in labor productivity. Hence, this paper proposes the following hypothesis:

*H*4: The DIG promotes CTFP by driving human capital.

## 3 Research design

### 3.1 Methods

To examine the impact of DIG development on CTFP, the basic regression model of the relationship between the DIG and CTFP is constructed, as shown in Equation (1):


(1) $$CTFP_{\mathrm{it}}=\alpha_{0}+\alpha_{1}DIG_{\mathrm{it}}+\alpha_{2}{\mathrm{Cont}}_{\mathrm{it}}+\varepsilon_{\mathrm{it}}$$

In Equation (1), *i* and *t* represent the region and year, respectively; *ε* is the error term; and α is the coefficient. CTFP represents the dependent variable, DIG represents the level of DIG development, and Cont represents all the control variables. The first hypothesis suggests that a one-unit change in DIG will result in $\alpha_{1}$ corresponding unit change in CTFP, assuming all other variables remain constant in Equation (1).

In addition to the total effect embodied in Equation (1), the DIG may have an indirect impact on CTFP through some mediating mechanisms. According to the research hypotheses above, the DIG may improve CTFP through technological innovation, the factor endowment structure and human capital. Therefore, the following mediating effect model is established in this paper:


(2) $$M_{\mathrm{it}}=\beta_{0}+\beta_{1}DIG_{\mathrm{it}}+\beta_{2}\mathrm{Con}t_{\mathrm{it}}+\varepsilon_{\mathrm{it}}$$

(3) $$CTFP_{\mathrm{it}}=\gamma_{0}+\gamma_{1}DIG_{\mathrm{it}}+\gamma_{2}M_{\mathrm{it}}+\gamma_{3}\mathrm{Con}t_{\mathrm{it}}+\varepsilon_{\mathrm{it}}$$

M represents the mediating variables, which are technological innovation (RD), the factor endowment structure (KL) and human capital (HC). The other variables are defined the same as in Equation (1). Based on hypotheses 2–4, the study initially assesses the impact of DIG on the mediating variables, as depicted in Equation (2). Significance of coefficient $\beta_{1}$ indicates a substantial influence of DIG on the mediating variables. Subsequently, both DIG and the mediating variables are integrated into the model, as illustrated in Equation (3). If coefficients $\gamma_{1}$ and $\gamma_{2}$ are significant, it suggests that DIG affects CTFP through intermediary variables.

### 3.2 Variables

#### 3.2.1 Dependent variable

When DEA is used to measure CTFP, the Luenberger productivity index can compensate for the shortcomings of the Malmquist index and Malmquist–Luenberger productivity index. The decrease in “bad” output and the increase in “good” output fit the farthest-point distance function. Therefore, this study selects a model that combines the SBM undesirable model containing undesirable output and the Luenberger productivity index to calculate CTFP.

For a specific $DMU_{0}\left(x_{0},y_{0}^{g},y_{0}^{b}\right)$ , Tone (29) proposed an SBM undesirable model including both “good” and “bad” outputs. This model solved the slack problem of input–output variables. The model is shown in Equation (4):


(4) $$\begin{array}{l} {{\vec{{\vec{S}}_{C}^{t}}}^{t}}_{c}\left(x_{k}^{t},y_{k}^{t},b_{k}^{t}\right)=\rho^{*}=\min\frac{1-\frac{1}{m}\sum_{i=1}^{m}\frac{s_{i}^{-}}{x_{i0}}}{1+\frac{1}{s_{1}+s_{2}}\left(\sum_{r=1}^{s_{1}}\frac{s_{r}^{g}}{y_{r_{0}}^{g}}+\sum_{r=1}^{s_{2}}\frac{s_{r}^{b}}{y_{r0}^{b}}\right)}\\ s.t.\{\begin{array}{l} x_{0}=X\lambda +s^{-}\\ y_{0}^{g}=Y^{g}\lambda -s^{g}\\ y_{0}^{b}=Y^{b}\lambda +s^{b}\\ \lambda \geq 0,s^{-}\geq 0,s^{g}\geq 0,s^{b}\geq 0 \end{array} \end{array}$$

where *t* is the period (2011–2020), *k* is 30 Chinese provinces, *s^g^* is the expected output shortage, and *s^−^* and *s^b^* represent the excess of inputs and the excess of undesirable outputs, respectively. The objective function ρ* is strictly decreasing with respect to *s^−^*, *s^g^*, and *s^b^*.

On the basis of Equation (4) and the ideas of Chambers et al. (30), the CTFP index is constructed from period t to period t + 1 (see Equation 5):


(5) $$\begin{array}{l} \mathrm{CTFP}\left(x^{t+1},y^{t+1},f^{t+1};x^{t},y^{t},f^{t}\right)={\left(\frac{{\vec{S}}_{C}^{t}\left(x^{t+1},y^{t+1},b^{t+1}\right)}{{\vec{S}}_{C}^{t}\left(x^{t},y^{t},b^{t}\right)}\times \frac{{\vec{S}}_{C}^{t+1}(x^{t+1},y^{t+1},b^{t+1}}{{\vec{S}}_{C}^{t+1}\left(x^{t},y^{t},b^{t}\right)}\right)}^{1/2}\\ =\frac{{\vec{S}}_{C}^{t+1}\left(x^{t+1},y^{t+1},b^{t+1}\right)}{{\vec{S}}_{C}^{t}\left(x^{t},y^{t},b^{t}\right)}\times {\left(\frac{{\vec{S}}_{C}^{t1}(x^{t+1},y^{t+1},b^{t+1}}{{\vec{S}}_{C}^{t+1}\left(x^{t+1},y^{t+1},b^{t+1}\right)}\times \frac{{\vec{S}}_{C}^{t}\left(x^{t},y^{t},b^{t}\right)}{{\vec{S}}_{C}^{t+1}\left(x^{t},y^{t},b^{t}\right)}\right)}^{1/2}\\ =TEC\;\left(x^{t+1},y^{t+1},b^{t+1};x^{t},y^{t},b^{t}\right)\times \mathrm{EFF}\;\left(x^{t+1},y^{t+1},b^{t+1};x^{t},y^{t},b^{t}\right) \end{array}$$

where EFF and TEC represent the changes in technical efficiency and technological progress from t to t + 1, respectively. If the values of CTFP, EFF and TEC are greater than 1, they mean CTFP growth, technical efficiency growth and technological progress improvement, respectively; if the values of CTFP, EFF and TEC are less than 1, they mean CTFP decline, technical efficiency deterioration and technological regression, respectively.

Three inputs and two outputs are selected for measuring CTFP: the three inputs are employed persons, the capital stock and total energy consumption, and the two outputs are desirable and undesirable outputs. The desirable output is regional GDP (13). To remove the effects of inflation, GDP is measured in 2010 constant prices. The undesirable output is the comprehensive environmental pollution index, which uses the entropy method to combine PM_2.5_ pollution with wastewater emissions, general solid waste production, sulfur dioxide emissions, nitrogen oxide emissions, and smoke dust emissions. Employed persons are measured as the number of employees at the end of the calendar year (12). The capital stock is the total existing capital resources of each province, and it is calculated by the perpetual inventory method (11). The equation is $K_{\mathrm{it}}=I_{\mathrm{it}}+\left(1-\delta_{\mathrm{it}}\right)K_{\mathrm{it}-1}$ , where *K_it_* represents the actual capital stock of province *i* in year *t* and *I_it_* represents fixed asset investment in province *I* in year *t*. In addition, considering the comparability problem, the total fixed asset investment in each province is deflated using the fixed asset investment price index, with 2010 as the base period. At the same time, the initial capital stock in 2010 is represented by dividing fixed asset investment by 10%, and the depreciation rate *δ_it_* is equal to 9.6% (31). Total energy consumption is the direct creator of environmental pollution and a major contributor to GDP growth (32).

#### 3.2.2 Independent variable

The measurement of the DIG draws on the method of Zhao et al. (33) and involves relevant indicators from five levels: the internet penetration rate, the number of internet-related employees, internet-related output, digital financial inclusion development, and the number of mobile internet users (see Table 1).

**Table 1:** Digital economy evaluation index system.

Secondary indicators	Tertiary indicators	Indicator explanation
Internet penetration rate	Internet users per 100 people	Reflects the internet penetration in each city
Number of internet-related employees	Proportion of employees in computer services and software	Reflects the degree of development of the digital economy
Internet-related output	Number of mobile Internet users	Reflects the degree of internet demand in each city
Digital financial inclusion development	China digital financial inclusion index	Reflects the degree of development of digital finance
Number of mobile internet users	Number of mobile phones per 100 people	Reflects the popularity of telecommunications

The five-level indicators in Table 1 are dimensionally reduced by the entropy method and condensed into a comprehensive DIG index. The adoption of the entropy method in this paper is motivated by the following reasons: Firstly, it eliminates the need for expert scoring or subjective valuation, thereby mitigating the influence of human bias. Secondly, it enables analysis of the correlation between indicators and adjustment of weights accordingly, thus enhancing the accuracy of comprehensive evaluation. The calculation process for the entropy method is illustrated in formulas (6)–(11). The comprehensive DIG index of each province from 2011 to 2020 is calculated; Table 2 presents the descriptive statistics of the DIG. The table shows that from 2011 to 2020, the level of DIG development was very different, and the level of DIG development showed an upwards trend over time. We take 2011 and 2020 as examples. In 2011, the top five provinces were Beijing, Shanghai, Guangdong, Zhejiang and Fujian, while the bottom five were Yunnan, Henan, Jiangxi, Gansu and Guizhou. In 2020, the top five provinces were Beijing, Shanghai, Zhejiang, Guangdong, and Tianjin, while the bottom five were Jilin, Hunan, Jiangxi, Hebei and Heilongjiang. Comparing 2011 and 2020, the level of DIG development of the top-ranked provinces changed slightly, while the level of DIG development of the bottom-ranked provinces changed greatly. These findings indicate that provinces with a good foundation for DIG development give full play to their own advantages and widen the gap with other provinces.


(6) $$x_{ij}=\frac{x_{ij}-\min\left\{x_{j}\right\}}{\max\left\{x_{j}\right\}-\min\left\{x_{j}\right\}}$$

Where $\max\left\{x_{j}\right\}$ is the maximum value of the indicator in all years; $\min\left\{x_{j}\right\}$ is the minimum value of the indicator in all years; $x_{ij}$ is the result of dimensionless.


(7) $$w_{ij}=\frac{x_{ij}}{\overset{m}{\underset{i=1}{\sum }}x_{ij}}$$

Where $w_{ij}$ represents the proportion of *j* indicators in year *i*.


(8) $$e_{j}=-\frac{1}{lnm}\overset{m}{\underset{i=1}{\sum }}\left(w_{ij}\times \ln w_{ij}\right)$$

Where $e_{j}$ represents the information entropy of the indicator.


(9) $$d_{j}=1-e_{j}$$

Where $d_{j}$ represents the information entropy redundancy.


(10) $$\varphi_{j}=\frac{d_{j}}{\overset{m}{\underset{i=1}{\sum }}d_{j}}$$

Where $\varphi_{j}$ represents the index weight.


(11) $$DIG_{i}=\overset{m}{\underset{j=1}{\sum }}\varphi_{j}\times w_{ij}$$

Where $DIG_{i}$ represents digital economy development level of each province.

**Table 2:** Descriptive statistics of the digital economy.

	2011	2012	2013	2014	2015	2016	2017	2018	2019	2020
Average	0.131	0.197	0.263	0.293	0.341	0.344	0.409	0.501	0.590	0.657
Min	0.077	0.139	0.196	0.233	0.280	0.287	0.352	0.428	0.504	0.556
Max	0.314	0.399	0.479	0.521	0.586	0.565	0.639	0.773	0.895	0.982
Stedv.	0.052	0.057	0.062	0.062	0.066	0.059	0.063	0.076	0.085	0.089

Calculated based on the entropy method combined with the index system in Table 1.

#### 3.2.3 Control variables

Referring to the literature (1–3, 32), this study controls for the following variables that may affect CTFP. (i) Economic development level (PGDP). This variable is expressed by *per capita* GDP, which is used to measure the impact of regional economic development on CTFP (9). (ii) Population agglomeration (POP). This variable is expressed by the population per unit area, and the square term of POP is introduced to investigate whether there is a non-linear relationship between population agglomeration and CTFP (12). (iii) Environmental regulation (ENVR). This variable is expressed by the proportion of environmental governance investment in GDP. It is used to measure the impact of the level of environmental regulation on CTFP (18). (iv) Economic openness (FDI). This variable is expressed by the proportion of the actual use of foreign direct investment in GDP and is denoted by FDI. It is used to examine the impact of foreign direct investment on CTFP (32). (v) Industrial structure (ST). This variable is expressed by the ratio of the added value of the secondary industry to the added value of the tertiary industry. It is used to verify the influence of the rationality of the industrial structure on CTFP (32).

#### 3.2.4 Mediator variables

Based on the theoretical analysis above, this paper selects the following mediator variables. (i) Technological innovation (RD). This variable is expressed by the ratio of R&D expenditure to GDP. It is mainly used to examine the impact of the level of expenditure on science and technology on CTFP (16). (ii) Factor endowment structure (KL). This variable adopts the ratio of the capital stock to the number of laborers in each province. Its purpose is to examine the influence of the rationality of factor endowments on CTFP to further optimize resource allocation (17). (iii) Human capital (HC). This variable is expressed by the *per capita* years of education (34). The calculation method is as follows: *per capita* years of education = (the number of illiterate people * 1 + the number of people with a primary school education * 6 + the number of people with a junior high school education * 9 + the number of people with a high school and technical secondary school education * 12 + the number of people with a college degree or above *16)/the total population older than 6.

#### 3.2.5 Data and statistical description

The data on the three inputs as well as GDP, wastewater emissions, general solid waste production, sulfur dioxide emissions, nitrogen oxide emissions, and smoke dust emissions are from the China Statistical Yearbooks (2012–2021), the China Energy Statistics Yearbooks (2012–2021) and the China Environmental Statistical Yearbooks (2012–2021). PM2.5 pollution concentration data are obtained from the International Geoscience Information Network Center of Columbia University and processed with ArcGIS 10.0 software (1, 32). The CTFP indicator is calculated by the CTFP model. The DIG indicator is from the China Statistical Yearbook and the Digital Finance Research Center of Peking University and is calculated by the entropy method. The data on other control variables are from the 2012–2021 China Statistical Yearbook and provincial statistical yearbooks. The descriptive statistics are shown in Table 3.

**Table 3:** Descriptive statistics of the variables.

Variable	Variable	Unit	Mean	SD	Min	Max
Input	Employed persons	10,000 persons	2703.55	2155.50	279.00	7150.25
	Capital stock	100 million	106786.50	90953.48	10512.23	298851.11
	Total energy consumption	10,000 tce	15200.83	12119.25	1601.00	41826.80
Desired output	GDP	100 million yuan	23589.36	18439.97	1519.23	93014.74
Undesired output	Environmental pollution comprehensive index	–	48883.38	40979.25	6557.08	181211.54
Dependent variable	CTFP	–	1.43	0.467	0.91	5.14
Independent variable	DIG	–	0.37	0.17	0.08	0.98
Control variables	PGDP	RMB	8920.77	13908.29	160.82	127760.7
	POP	Person/ square kilometer	466.50	697.17	7.89	3922.86
	ENVR	%	1.32	0.77	0.22	4.24
	FDI	%	0.02	0.02	0.00	0.08
	ST	%	0.93	0.33	0.06	1.90
Mediator variables	RD	%	1.62	1.07	0.33	6.01
	KL	RMB/person	42.77	17.39	19.16	116.65
	HC	year	9.32	0.89	7.68	12.78

“–” means there is no unit, and the result is calculated by the entropy method.

## 4 Results and discussion

### 4.1 Measurement of CTFP and the decomposed effects

MaxDEA 6.16 software is combined with Equation (5) to measure the CTFP and decomposed effects of 30 Chinese provinces from 2011 to 2020. The results are provided in Table 4.

(1) Changes in and decomposed effects on China’s CTFP. Table 4 shows that from 2011 to 2020, the average growth rate of the CTFP index was 7.82%, of which EFF contributed 0.18% and TEC contributed 7.77%. From the perspective of the decomposed effects, both EFF and TEC could improve China’s CTFP, but TEC was the main source of power.
(2) The trend and decomposition of CTFP in different regions. From 2011 to 2020, the average growth rate of the CTFP index in the eastern region was 9.48%, of which the contribution rate of EFF was 0.84% and the growth rate of TEC was 8.63%. From 2011 to 2020, the average growth rate of the CTFP index in the central region was 7.05%, of which the contribution rate of EFF was 0.13% and the contribution rate of TEC was 6.97%. From 2011 to 2020, the average growth rate of the CTFP index in the western region was 6.74%, of which the contribution rate of EFF was −0.45% and the contribution rate of TEC was 7.48%. In general, the growth rates of CTFP in the eastern, central and western regions decreased in turn. The EFF of the eastern and central regions had a slight effect on improving their CTFP, while the TEC of the western region had a restraining effect. TEC was the main driving force behind the improvement in CTFP in the three regions.

**Table 4:** Descriptive statistical results of CTFP and the decomposed effects.

Regions/Variables		2011–2012	2012–2013	2013–2014	2014–2015	2015–2016	2016–2017	2017–2018	2018–2019	2019–2020	Avg.
China	CTFP	1.077	1.085	1.049	1.079	1.077	1.039	1.129	1.059	1.110	1.078
	TEC	1.071	1.071	1.057	1.084	1.081	1.035	1.123	1.089	1.088	1.078
	EFF	1.006	1.013	0.993	0.996	0.998	1.004	1.005	0.978	1.021	1.002
ERC	CTFP	1.096	1.081	1.069	1.104	1.101	1.058	1.140	1.051	1.153	1.095
	TEC	1.094	1.083	1.063	1.108	1.099	1.047	1.148	1.057	1.078	1.086
	EFF	1.001	0.998	1.005	0.997	1.003	1.011	0.994	0.995	1.070	1.008
CRC	CTFP	1.071	1.105	1.020	1.053	1.065	1.043	1.113	1.084	1.081	1.070
	TEC	1.061	1.075	1.054	1.083	1.060	1.020	1.128	1.074	1.071	1.070
	EFF	1.010	1.028	0.968	0.975	1.005	1.022	0.986	1.009	1.009	1.001
WRC	CTFP	1.064	1.074	1.050	1.072	1.060	1.018	1.130	1.050	1.088	1.067
	TEC	1.054	1.057	1.052	1.059	1.079	1.033	1.096	1.133	1.110	1.075
	EFF	1.009	1.017	0.999	1.011	0.987	0.985	1.030	0.938	0.982	0.995

Data source: Calculated using MaxDEA 6.16 software.

### 4.2 Benchmark regression

In this paper, STATA 15.0 software is used for quantitative regression. To avoid heteroscedasticity, logarithmic processing is adopted for each variable. The variance inflation factor is employed to examine the presence of multicollinearity among the variables. The average value of the variance inflation factor is 1.54, significantly below 10, indicating the absence of multicollinearity. Equation (1) is estimated by using ordinary least squares (OLS), fixed effects (FE), and random effects (RE) models, which are shown in Table 5. The F test and Hausman test are performed for OLS, FE and RE models to select a suitable panel model. The results of the *F*-test are *F*(29, 263) = 6.11, Prob>*F* = 0.0000, and the results of the Hausman test are chi2(8) = 55.64, Prob>*F* = 0.0000. All of these results show that the FE model is the best model among the three. Therefore, the analysis of the results is conducted based on the results of the FE model.

**Table 5:** Benchmark regression results.

Variable	Dependent variable: CTFP		
	(1)	(2)	(3)
Estimation method	OLS	RE	FE
Constant	−0.050 (−0.34)	0.052 (0.23)	7.456** (2.18)
LnDIG	0.344*** (12.99)	0.330*** (13.89)	0.319*** (12.37)
LnPGDP	0.056*** (5.40)	0.045*** (4.31)	0.041*** (3.69)
LnPOP	0.061 (1.40)	0.063 (0.78)	−3.030** (−2.56)
LnPOP^2^	−0.004 (−0.99)	−0.004 (−0.51)	0.299*** (2.83)
LnENVR	−0.035* (−1.72)	−0.059*** (−2.60)	−0.065*** (−2.65)
FDI	−1.936** (−2.25)	−3.074*** (−3.19)	−3.131*** (−2.96)
LnST	0.081*** (2.93)	0.033 (1.10)	0.011 (0.34)
R2	0.584	0.675	0.687
OBS	300	300	300
F statistic	*F*(29, 263) = 6.11, Prob>F = 0.0000		
Hausman tests	chi2(8) = 55.64, Prob>F = 0.0000		

The values in parentheses are *t*-values; *, **, and *** indicate that the estimated coefficients are significant at the 10, 5, and 1% levels, respectively. OLS, RE, and FE represent mixed effects estimation, random effects estimation, and fixed effects estimation, respectively. The asterisks in the following tables have the same meaning as the notes here.

As shown in Table 5, column (3) shows that the DIG has significantly improved the CTFP of Chinese provinces. The impact coefficient is 0.319 and is significant at the 1% level. With its continuous and rapid development, the DIG has continuously penetrated various fields through information technology, reduced the massive emission of environmental pollutants, improved the efficiency of utilization in the production sector, and thus improved CTFP. In addition, the underlying reason for the direct and positive impact of the DIG on CTFP is that the characteristics of replicability and shareability of the DIG make data elements overcome the traditional law of diminishing marginal returns (35). The environmentally friendly features of consumption and low pollution emissions, as well as the features of low cost and high returns, have a positive effect on CTFP. This conclusion verifies that hypothesis 1 is correct. Regarding the other control variables, the coefficient of PGDP is positive and significant at the 1% level, indicating that the increase in PGDP is conducive to improving CTFP. The first-order coefficient of POP is negative, the second-order coefficient is positive, and both are significant. These results indicate that there is a U-shaped curvilinear relationship between population agglomeration and CTFP. In the initial stage, population agglomeration consumes more resources and energy and produces many environmental pollutants. When population agglomeration reaches a certain level, it can improve labor productivity, realize economies of scale, improve the utilization efficiency of resources and energy, and lead to an improvement in CTFP (36). ENVR inhibits an improvement in CTFP. The reason for this result may be that most enterprises across China passively accept environmental supervision. After raising production costs to meet the requirements of environmental regulatory policies, they must reduce their production input under capital pressure and reduce their investment in green technology. Technological innovation requires more investment and a long period of time to generate benefits. As a result, the cost of environmental regulation is greater than the benefits obtained, and CTFP declines (6). FDI negatively and significantly affects CTFP. One possible reason is that the entry of FDI may transfer a large amount of energy-intensive manufacturing or pollution-intensive industries to China, causing a considerable amount of environmental pollution and making the host country a “pollution paradise.” It is also possible that enterprises in various regions of China become technologically dependent on foreign technology, inhibiting the independent innovation of Chinese enterprises (32). The coefficient of ST is positive but not significant. There is no evidence to show that ST has a significant impact on CTFP. One possible reason is that there is a non-linear relationship or other influence between ST and CTFP.

### 4.3 Mediating effect test

The previous estimation results confirm that the DIG plays a significant role in improving CTFP. Consistent with the previous theoretical analysis, this paper further explores the mechanisms of the impact of the DIG on CTFP through technological innovation (RD), the factor endowment structure (KL) and human capital (HC). The results are presented in Table 6.

**Table 6:** Mediating effect test results.

Variable	Technological innovation	Comprehensive total factor productivity	Factor endowment structure	Comprehensive total factor productivity	Human capital	Comprehensive total factor productivity
	lnRD(1)	lnCTFP(2)	lnKL(3)	lnCTFP(4)	lnHC(5)	lnCTFP(6)
Constant	2.512 (0.94)	6.955** (2.06)	13.123*** (3.03)	6.021* (1.75)	2.857*** (5.56)	5.415 (1.51)
lnDIG	0.107*** (5.28)	0.298*** (11.09)	0.076** (2.33)	0.311*** (12.02)	0.043*** (11.04)	0.289*** (9.28)
lnRD		0.199** (2.56)				
lnKL				0.109** (2.27)		
lnHC						0.714** (1.75)
Controls	Yes	Yes	Yes	Yes	Yes	Yes
R^2^	0.270	0.694	0.081	0.693	0.573	0.690
N	300	300	300	300	300	300

#### 4.3.1 Technological innovation effect

Column (1) reveals that the coefficient of the effect of the DIG on technological innovation is positive and significant at the 1% level. This result indicates that DIG development promotes technological innovation. Column (2) shows that the coefficient of the effect of DIG on CTFP is positive and significant at the 1% level. Additionally, the coefficient of the effect of technological innovation on CTFP is positive and significant at the 5% level. These results show that there is a mediating effect in which the DIG promotes CTFP through technological innovation, which is consistent with the analysis in Hypothesis 2. One possible reason for this conclusion is that the powerful financing ability of the DIG can provide a financial guarantee for the technological innovation of enterprises. Additionally, the green technological innovation of enterprises can reduce environmental pollution and improve CTFP, as supported by the studies conducted by Cheng et al. (37) and Zhao et al. (28).

#### 4.3.2 Factor endowment structure effect

The estimates of the factor endowment structure effect are presented in Column (3) of Table 6. The coefficient of the effect of the DIG on the factor endowment structure is estimated to be 0.076 and is significant at the 5% level. This result implies that the DIG has a positive impact on the factor endowment structure. Column (4) shows that the coefficient of the DIG on CTFP is positive and significant at the 1% level. Additionally, the coefficient of the effect of the factor endowment structure on CTFP is positive and significant at the 5% level. These results show that there is a mediating effect in which the DIG promotes CTFP through the factor endowment structure. This conclusion verifies that hypothesis 3 is correct. One possible reason is that under the background of the DIG, each region will monitor the factor endowment in each detail and evaluate the local factor endowment structure. Then, industries will adjust based on the structure in a timely manner, open up the industrial chain, realize the connection between the bottom production factors and the top final product demand, and realize the upgrading of the factor endowment structure. The whole region will combine production factors and the industrial structure to meet market demand. In this process, the optimal allocation of the factor endowment structure will be realized through the DIG, and CTFP will ultimately be improved (38).

#### 4.3.3 Human capital effect

The estimates of the human capital effect are presented in Column (5) of Table 6. The coefficient of the effect of the DIG on human capital is estimated to be 0.043 and is significant at the 1% level. This result implies that the DIG has a positive impact on human capital. Column (6) shows that the coefficient of the effect of the DIG on CTFP is positive and significant at the 1% level. Additionally, the coefficient of the effect of human capital on CTFP is positive and significant at the 5% level. These results show that there is a mediating effect in which the DIG promotes CTFP through human capital. This conclusion verifies that hypothesis 4 is correct. The primary reason behind these findings is that online and offline agglomeration, information network sharing and the efficient matching of big data algorithms can quickly realize a transformation of human capital from agglomeration to sharing and improve the level of human capital agglomeration. The improvement in the human capital level will prompt people to raise their requirements for the quality of the surrounding environment, prompting a transition to green consumption behavior and the green production behavior of enterprises (39). This transition will produce a reduction in environmental pollution and, in turn, a promoting effect that further improves CTFP.

### 4.4 Endogeneity tests

Considering the potential endogeneity problems in the model, this paper further uses the instrumental variable estimation method to test the impact of the DIG on CTFP. The selection of instrumental variables should meet the two conditions of being “independent of dependent variables” and “related to endogenous variables” (38, 40). (1) Referring to Zhao et al. (28), this paper takes the DIG (L.DIG) as the instrumental variable. The results of the two-stage regression are presented in columns (1) and (2) of Table 7. The F-statistic values of the weak instrumental variable tests in the first stage are all significant at the 1% level, indicating that the estimation results of the instrumental variables are valid. The estimation results of the second stage reveal that the coefficients of L.DIG align closely with the findings in the baseline regression results, reflecting that the impact of the DIG on CTFP is robust and credible. (2) With reference to relevant literature (41, 42), this paper additionally incorporates two other instrumental variables of the digital economy: the interaction term obtained by multiplying the number of fixed telephones per 100 individuals in 1984 with the national information technology service income (Lntel), and the interaction term derived from multiplying the number of post offices per million individuals in 1984 with the national information technology service income (Lnpost). The results are presented in columns (3) and (4) of Table 7. The coefficient of the Lntel’s effect on CTFP exhibits a positive and statistically significant association at the 1% level, as does the coefficient of the Lnpost’s effect on CTFP. These findings align with benchmark regression results, indicating that the effect of the DIG on CTFP remains robust.

**Table 7:** Endogeneity test results.

Variable	(1)	(2)	(3)	(4)
	First stage	Second stage	LnCTFP	LnCTFP
LnDIG			0.354*** (7.64)	0.325*** (13.73)
L.LnDIG	0.747*** (44.68)	0.300*** (10.06)		
LnTel			0.281*** (14.46)	
LnPost				0.268*** (13.24)
Control variables	YES	YES	YES	YES
R2	0.959	0.633	0.705	0.701
N	270	270	300	300
*F*-value	775.33			

### 4.5 Robustness tests

#### 4.5.1 Quantile regression method

Quantile regression was conducted to examine the robustness of the findings. The results of quantile regression at 75 and 90% are presented in columns (1) and (2) of Table 8, respectively. Notably, all coefficients associated with the digital economy in quantile regression passed the significance test level of 1%, indicating that the impact of digital economy on CTFP remains robust.

**Table 8:** Robustness test results.

Variable	(1)	(2)	(3)	(4)
	LnCTFP(Quantile = 0.75)	LnCTFP(Quantile = 0.90)	LnCTFP	LnTEC
LnDIG	0.332*** (5.54)	0.341*** (2.70)	0.354*** (7.64)	0.325*** (13.73)
L.LnCTFP			0.122* (1.73)	
Control variables	YES	YES	YES	YES
R2			0.657	0.729
N	300	300	270	300

#### 4.5.2 Replacing the explained variable

This paper performs a robustness test by substituting the explained variable, CTFP. First, the first-order lag of CTFP (L.LnCTFP) is added to the measurement model as an explanatory variable for re-estimation. The results are presented in Column (3) of Table 8. The coefficient of the effect of the DIG on CTFP is positive and significant at the 1% level, and the coefficient of L.LnCTFP on CTFP is positive and significant at the 10% level. This conclusion is consistent with the results of the basic regression. Second, the TEC index, the decomposed index of CTFP, is used as a proxy variable for empirical testing, and the results are presented in Column (4) of Table 8. The coefficients of the DIG are positive and significant; thus, they are in line with the findings in the baseline regression results. These tests suggest a certain degree of robustness and reliability in the basic regression results.

### 4.6 Heterogeneity analysis

To investigate the heterogeneity between the DIG and CTFP in regional development, the research sample is divided into three regions according to the standards of the National Bureau of Statistics of the People’s Republic of China: eastern region of China (ERC), central region of China (CRC), and western region of China (WRC). The heterogeneity test results are shown in Table 9. Columns (1), (2), and (3) reveal that the regression coefficients of the DIG are 0.495, 0.358, and 0.261, respectively, indicating that the impact of the DIG on CTFP decreases in turn from the ERC to the CRC and WRC. Meanwhile, the regression coefficients of the DIG are all positive and significant at the 1% level. These results indicate that the DIG can significantly improve the level of CTFP in the three major regions of China.

**Table 9:** Heterogeneity analysis results.

Variable	ERC(1)	CRC(2)	WRC(3)
constant	20.961 (1.09)	7.741 (0.72)	9.988** (2.44)
LnDIG	0.495*** (7.56)	0.358*** (6.72)	0.261*** (5.39)
Control	YES	YES	YES
R2	0.712	0.779	0.693
N	110	80	110

## 5 Conclusion and policy recommendations

### 5.1 Conclusion

The DIG has become one of the important ways to drive high-quality economic development in China, and CTFP is an important manifestation of high-quality economic development. The impact of the DIG on CTFP and the transmissions mechanisms have not been fully discussed. For this reason, this paper constructs a new undesirable output to measure the CTFP of China and empirically examines the effect of the DIG on CTFP and the mechanisms of this effect. Panel data covering 30 Chinese provinces and spanning from 2011 to 2020 are employed for analysis. The main conclusions drawn are as follows:

First, CTFP increased by an average of 3.9% in China. Technical efficiency contributed −1.1%, and the contribution rate of technological progress was 5.0%. Technological progress was the main source of total factor productivity growth.

Second, from a regional perspective, the growth rates of CTFP in the ERC, CRC, and WRC decreased in turn. The technical efficiency of the ERC and CRC had a slight effect on improving their CTFP, while the technical efficiency of the WRC had a restraining effect. Technological progress was the main driving force behind the improvement in CTFP in the three regions.

Third, the DIG had a positive and significant impact on CTFP, and this impact was significant in the ERC, CRC, and WRC. These findings remain consistent with the previous conclusion after endogeneity tests and various robustness tests.

Finally, mechanism tests suggest that the promoting effect of the DIG on CTFP can be attributed to three main effects: technological innovation, the factor endowment structure and human capital.

### 5.2 Policy recommendations

Several policy implications can be drawn from the conclusions above.

First, the governments of various regions in China should clearly see the differences between regions and formulate development strategies that correspond to them to promote haze governance between regions as well as CTFP convergence and balance. They should also make efforts to build regional comparative advantages, give full play to the orderly and free flow of various elements, and form a regional development pattern with benign interactions between regions. For regions with a high level of digital economy development, they should capitalize on their advantages by allocating sufficient research and development funds to foster the advancement of digital technology, thereby expediting the evolution of hardware infrastructure. Simultaneously, it is imperative for them to establish a core data platform that serves as the central engine driving data-centric elements. For regions with a lower level of digital economy development, the government should initially provide substantial foundational data to facilitate the growth of digital technology and stimulate its positive impact on traditional markets. Additionally, authorities should enhance support for such cities through research and development subsidies, preferential tax rates, and other measures aimed at guiding their sustainable development.

Second, all localities should use digital transformation to drive the transformation of traditional modes of production and governance as well as traditional ways of life, realize energy conservation, emission reduction and resource utilization efficiency improvement, fully release the role of the DIG in promoting green economic efficiency, and achieve high-quality economic development in China. The formulation of relevant policies and systems, such as the implementation of open market access policies, should be undertaken by all regions. It is essential to ensure compliance while striving for maximum cross-regional and cross-industry flow of production factors in order to achieve a more extensive allocation of resources. Additionally, establishing a production factor information sharing platform can effectively reduce information asymmetry by providing comprehensive data on talent demand, market demand, investment opportunities, etc. This will contribute to the creation of a favorable information environment in China that promotes the realization of digital economy system dividends. Consequently, it will facilitate better matching between production factors and market demand while enhancing resource allocation efficiency.

Third, the government should pay attention to optimizing the structure of foreign investment. While expanding the scale of foreign investment, the government should strengthen the assessment of the quality of foreign investment, guide the transfer of foreign investment to new high-tech industries, reduce investment in high-pollution, high-emission and high-energy-consuming industries, give full play to the “pollution halo effect” of foreign direct investment in various regions of China, realize more technology spillover effects, and improve total factor productivity. On one hand, it is imperative for all regions to enhance environmental standards, refine the negative list for foreign investment access, optimize the structure of attracting foreign investment, and augment the proportion of technology-intensive foreign-funded enterprises. Simultaneously, relevant departments should bolster supervision to curb rent-seeking activities, rigorously regulate environmental standards for attracting FDI, curtail the entry of high-energy consumption and highly polluting foreign enterprises, thereby achieving the objective of enhancing the quality of attracting foreign investment while stimulating industrial upgrading and technological innovation in existing foreign investments.

Finally, all localities should improve the quality and skills of human capital in society as a whole, especially the cultivation of cross-border, integrated and practical talent under the “industry-university-research” system. All regions should also actively cultivate high-tech industries and knowledge-technology-intensive industries and realize the overall transformation of the industrial chain with digital technology. Digital technology should be used to improve the technological innovation system, enhance the independent innovation capabilities of market entities, and promote the green transformation and development of China’s economy. The government should enhance investment in education, technology research and development, and other related fields, while reallocating more fiscal expenditure toward education and scientific research. This will foster the cultivation of a greater number of innovative talents, enabling them to assume leadership roles as well as exert influence over innovation processes, thereby elevating the level of regional innovation. Simultaneously, it is imperative to augment investment in innovative human capital by offering higher wages to professionals engaged in higher education compared to ordinary employees. This will attract a larger pool of innovative talents while establishing a mechanism for joint talent training within the region to ensure effective flow and allocation.

## Data availability statement

The original contributions presented in the study are included in the article/supplementary material, further inquiries can be directed to the corresponding author.

## Author contributions

DM: Conceptualization, Formal analysis, Funding acquisition, Investigation, Software, Writing – original draft, Writing – review & editing. GL: Conceptualization, Data curation, Funding acquisition, Investigation, Methodology, Writing – original draft, Writing – review & editing.
